# Formation of the Cu+Nb Interlayer in the Inconel 718/Ti6Al4V Multi-Material Obtained by Selective Laser Melting

**DOI:** 10.3390/ma17235801

**Published:** 2024-11-26

**Authors:** Arseniy Repnin, Evgenii Borisov, Anatoly Popovich

**Affiliations:** Institute of Machinery, Materials, and Transport, Peter the Great St. Petersburg Polytechnic University (SPbPU), Polytechnicheskaya, 29, 195251 Saint Petersburg, Russia

**Keywords:** additive manufacturing, selective laser melting, multi-materials, interlayer, Inconel 718/Ti6Al4V

## Abstract

This study examines the Inconel 718/Ti6Al4V multi-material with a Cu and Nb interlayer produced by SLM. To achieve this, it is necessary to investigate the microstructure, the chemical and phase composition, and the hardness of the interfacial zone in the multi-material samples. Furthermore, it is necessary to determine the impact of interlayer utilization on the mechanical properties of multi-material samples. The investigation showed that the formation of island macro-segregation was observed in all interfacial zones of the multi-material samples. The interfacial zones, Ti6Al4V/Nb and Cu/Inconel 718, exhibited a relatively sharp transition in the chemical composition. In contrast, the Cu/Nb interfacial zone exhibited a gradual transition. The results of the chemical composition study indicated that the width of the Nb/Cu transition zone was approximately 700 μm. No new phases were identified in the production of the multi-material samples. The typical phases were present in the alloy zone, as well as in the Nb/Cu interfacial zone. During the transition from the Ti6Al4V zone to the Inconel 718 zone through the Nb and Cu zones, the average microhardness values changed as follows: 270 → 190 → 120 → 300 HV. The ultimate tensile strength values for the multi-material samples reached 910 MPa.

## 1. Introduction

Products with a multi-material structure exhibit a number of advantages over products with a homogeneous structure [1]. The current level of technological advancement in manufacturing enables the production of such products with complex geometries [2]. This is primarily achieved through the utilization of novel technologies [3]. The constraints of conventional technologies prevent the full realization of the capabilities of multi-materials [4]. Nevertheless, the utilization of advanced manufacturing technologies, such as additive manufacturing (AM), offers a promising solution for the successful production of multi-materials with complex geometries [5]. The different types of AM have the capability of producing multi-materials [6]. For example, selective laser melting (SLM) enables the successful production of multi-materials from metals and alloys [7]. Currently, research is being conducted on a range of multi-materials obtained by SLM: steel/copper alloys [8,9,10,11], stainless steels/heat-resistant nickel alloys [12,13,14], heat-resistant nickel alloys/copper alloys [15,16,17,18,19,20,21]. For example, in one of their studies, Tan C. et al. investigated the MS1/Cu multi-material obtained by SLM (where MS1 is maraging steel) [22]. An interfacial zone with a width of 30–40 µm was observed in the multi-material. The interface exhibited good fusion bonding with no defects. Tensile fracture occurred in the copper zone and not in the interfacial zone. The multi-material samples demonstrated higher strength properties than copper. Another important study by the same group of scientists was their work on W/Cu multi-materials obtained by SLM [23]. These kinds of multi-materials can be used as plasma-facing materials in fusion reactors. The results showed that a significant number of W particles were present in the interfacial zone, which was associated with the high cooling rate of copper. The interfacial zone of 50–80 μm has intense Marangoni convection within it.

The possibility of creating multi-materials from non-weldable alloys (alloys with metallurgical incompatibilities), including titanium alloys and heat-resistant nickel alloys [24,25,26], represents a remarkable prospect for improvement within the field of materials engineering. The significance of this type of multi-material is particularly evident in the aerospace industry [27,28,29]. The principal concept of this approach is to reduce the weight of products using a multi-material structure. The regions of the multi-material operating at elevated temperatures will be composed of a heat-resistant nickel alloy, while the regions operating at moderate temperatures will consist of a titanium alloy [30,31]. However, the fabrication of this type of multi-material product presents a number of challenges, including the formation of pores, cracks, non-melting, etc. [21].

In their paper, M. G. Scaramuccia et al. examined the multi-material Inconel 718/Ti6Al4V, which was obtained by SLM [21]. The authors observed the formation of cracks in this multi-material, which could be attributed to the presence of the brittle intermetallic compound (IMC) Ti_2_Ni when the amount of Inconel 718 exceeded 20 wt.%. To reduce the number of defects, the researchers suggested utilizing a gradient transition from Ti6Al4V to Inconel 718. In his doctoral thesis, Q. Li investigated a series of multi-materials produced by SLM, including a titanium α + β alloy (Ti6Al4V), Invar (FeNi36), and Cu10Sn [32]. The researcher observed that the presence of titanium in the Ti6Al4V alloy and nickel in the Invar alloy resulted in metallurgical incompatibility, leading to the formation of brittle IMCs such as TiNi_3_ and Ti_2_Ni. Furthermore, the interfacial zone of the Ti6Al4V/Invar multi-material contained FeNi and CuNi IMCs with different mechanical properties, causing the formation of cracks. Moreover, the presence of other IMCs, such as TiFe, was observed in the interfacial zone, with an impact on the microstructure and mechanical properties.

It can be observed that, in multi-materials produced by AM, in which one of the elements is a titanium-based alloy, the formation of cracks is evident in the interfacial zone. The formation of such cracks may be attributed to the influence of phase composition. To reduce this impact, interlayers are utilized in the production of multi-materials in both conventional technologies and AM [31,32,33,34]. Onuike et al. examined the multi-material Inconel 718/Ti6Al4V produced by direct laser deposition (DED) [33]. Three types of multi-material samples were considered: the build of Inconel 718 on Ti6Al4V, a gradient composition change from Inconel 718 to Ti6Al4V, and the utilization of an interlayer between the Inconel 718 and Ti6Al4V. The Ti6Al4V and Inconel 718 exhibited different thermal properties, and the formation of brittle IMCs at the interface between the two alloys was observed. This led to the development of delamination. To eliminate this defect, the authors used a composite interlayer, which was a mixture of vanadium carbide (VC), Ti6Al4V, and Inconel 718. This approach allowed for the production of a defect-free Inconel 718/Ti6Al4V multi-material. X-ray structural analysis revealed the presence of Cr_3_C_2_ phases. The composite interlayer enhanced the bond strength by reducing the formation of TiNi_3_ and Ti_2_Ni, and it mitigated thermal stresses in the interfacial zone.

In a series of papers, Xu G. and colleagues investigated a number of multi-materials obtained by DED: Ti6Al2Zr1Mo1V/Inconel718 + Nb and Cu interlayer [34], Ti6Al4V/Inconel718 + Ta and Cu interlayer [35], and Ti6Al4V/Inconel625 + Cu and V interlayer [36]. The authors demonstrated that the Ti6Al2Zr1Mo1V/Inconel718 multi-material with the Nb + Cu interlayer was free of macroscopic cracks and defects due to the effective restraint of Ti-Ni phase formation. The results of the microhardness tests demonstrated that the Nb/Cu interlayer was capable of facilitating a smooth transition from Ti6Al2Zr1Mo1V to Inconel 718 [34]. In the Ti6Al4V/Inconel718 multi-material with the Ta + Cu interlayer, the Ta/Cu interfacial zone exhibited no visible cracks or gas pores. The phase composition of this zone was as follows: α-Ti + β-Ti + β-Ta, β-Ta + γ-Cu + Ti2Cu + TiCu, and γ-Ni + γ-Cu + Cr2Ta + Laves phase. The formation of the Ta/Cu interfacial zone effectively prevented the formation of brittle IMCs such as Ti-Ni and reduced the cracking sensitivity of the multi-material. The formation of brittle IMCs such as Ti-Cu and Cr-Ta in the Ta/Cu interfacial zone was attributed to the diffusion of elements between the interlayers [35]. The utilization of the Cu + V interlayer in the Ti6Al4V/Inconel625 multi-material has been demonstrated to effectively prevent the formation of IMCs such as Ti-Ni [36].

A review of the literature regarding the production of multi-materials based on non-weldable alloys by SLM reveals that such multi-materials can be utilized in a broad range of possible applications. However, the manufacturing of such multi-materials gives rise to specific metallurgical issues, including the formation of pores, cracks, non-melting, etc., which present challenges in their production. These issues can be mitigated through the formation of an interlayer comprising metals and alloys. This approach has been successfully implemented in conventional technologies and DED. The Inconel 718/Ti6Al4V multi-material is of significant potential for utilization in the aerospace industry. Nevertheless, the fabrication of this multi-material presents certain challenges due to the non-weldability of the two alloys. Limited research has been conducted on the topic of the formation of an interlayer in the Inconel 718/Ti6Al4V multi-material obtained by SLM. This highlights the importance of conducting further studies in this area. This study examines the Inconel 718/Ti6Al4V multi-material with a Cu and Nb interlayer produced by SLM. For this purpose, it is necessary to examine the microstructure, the chemical and phase composition, and the hardness in the interfacial zones of the multi-material samples, in order to determine the impact of utilizing interlayers on the mechanical properties of the multi-material samples.

## 2. Materials and Methods

### 2.1. Starting Materials, L-PBF Process Parameters, and Inconel 718/Ti6Al4V Multi-Material Samples

The selected materials, Inconel 718 and Ti6Al4V, facilitate the realization of the suggested approach to performance enhancement, as outlined in the literature review. This approach involves the segmentation of the parts into distinct zones: with composition providing heat resistance and composition reducing mass. The Inconel 718 alloy exhibits high strength at elevated temperatures and good heat resistance [37]. The Ti6Al4V alloy is lightweight and exhibits high strength characteristics [38]. Furthermore, both alloys are widely utilized in SLM. The aforementioned considerations indicate that the Inconel 718/Ti6Al4V multi-material may have great prospects for the aerospace industry.

To obtain the Inconel 718/Ti6Al4V multi-material samples, it is proposed that an interlayer of Cu and Nb be employed. However, instead of pure Cu, the heat-resistant bronze alloy CuCr1Zr (0.5–1% Cr, 0.1–0.3% Zr, bal. Cu) was used. This alloy comprises approximately 98% Cu; therefore, for convenience in this paper, the interlayer will be referred to as Cu. It is also noteworthy that the alloy exhibits enhanced strength and heat resistance compared to pure Cu, which is a favorable property for its proposed application in the aerospace industry. It may be reasonably assumed that the presence of a minor quantity of alloying elements will not have a significant impact on the quality of the multi-material. The particle size distribution analysis indicates a similarity in the particle size (Table 1). The exception is Nb powder, which has a coarser particle size distribution. The discrepancy in the particle size distribution may result in the formation of a layer with certain disturbances during the printing process.

An SLM machine, the 3DLAM Mini (3DLAM LLC, St. Petersburg, Russia), was selected to produce the Inconel 718/Ti6Al4V multi-material samples. The samples were manufactured in an argon atmosphere on a titanium build platform. Modifications were made to the powder feeding device and to the distribution system for the second material, including the addition of a hopper, to facilitate the creation of multi-material samples on 3D printer. The process parameters for the fabrication of the multi-material samples are presented in Table 2. The parameters were selected based on the equipment used and an analysis of the literature on these metals and alloys [39,40,41,42].

**Figure 1:**
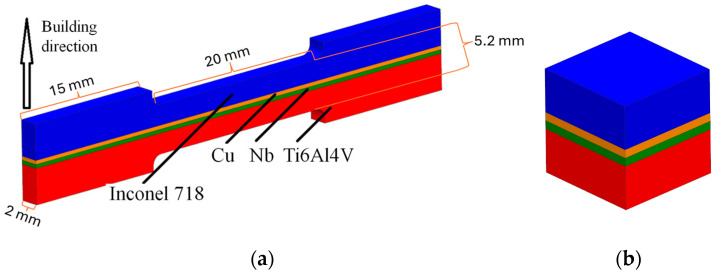
The geometry of the multi-material samples: (**а**) tensile test sample; (**b**) sample for metallographic study.

The metallographic and tensile test samples are shown schematically in Figure 1. The distribution of metals in the interlayers was as follows: 500 µm Cu + 500 µm Nb (according to the 3D model). The fabrication of multi-material samples was conducted in the following sequence: Ti6Al4V was printed first (approximately 2 mm), followed by Nb (500 μm), Cu (500 μm), and finally Inconel 718 (approximately 2 mm). The production of multi-materials on the modified 3DLam Mini machine allows the material to be changed in the main powder tank and in the multi-material tank during the printing process, with a minimal delay, without opening the working chamber of the machine. This feature makes it possible to produce multi-material samples from more than two alloys.

### 2.2. Characterization

A Leica DMi8 M optical microscope (Leica Microsystems, Wetzlar, Germany) was used to analyze defects and investigate the microstructure in the interfacial zone of Inconel 718/Ti6Al4V multi-material samples. The following etchants were utilized for the multi-material samples: a solution comprising 83% distilled water, 14% HNO_3_, and 3% HF, and a solution comprising 100 mL of distilled water, 11 mL of Na_2_SO_4_ solution, and 40 g of K_2_S_2_O_5_. A Mira 3 scanning electron microscope (TESCAN, Brno, Czech Republic) with an energy-dispersive X-ray spectroscopy module was used to investigate the chemical composition and to conduct fractographic analysis of the multi-material samples. The phase composition of the multi-material samples was analyzed using a Rigaku SmartLab X-ray diffractometer with CuKa radiation (Rigaku Corporation, Tokyo, Japan). For phase analysis, this equipment offers the possibility of X-ray microdiffraction with a beam width of 100 µm. The utilization of microdiffraction enables the direct investigation of the interlayer between two alloys. The Vickers MicroMet 5101 microhardness tester (Buehler Ltd., Lake Bluff, IL, USA) was used to analyze the microhardness in the multi-material samples. The mechanical testing of multi-material samples was carried out using a universal uniaxial floor-mounted testing machine (Zwick/Roell Group, Ulm, Germany).

## 3. Results and Discussion

### 3.1. Porosity and Microstructure of the Inconel 718/Ti6Al4V Multi-Material Samples

**Figure 2:**
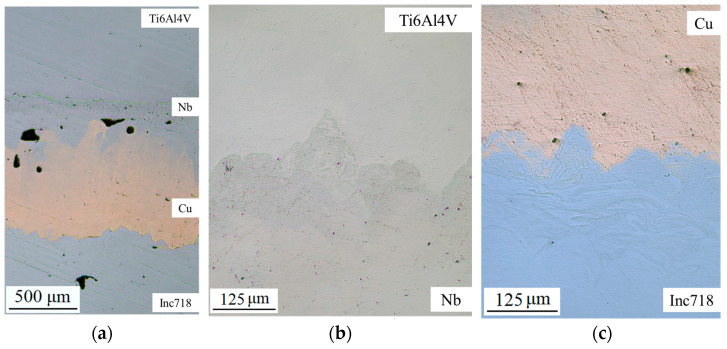
The defect analysis in the interfacial zones of the Inconel 718/Ti6Al4V multi-material sample: (**a**) the Ti6Al4V/Nb interfacial zone; (**b**) the Nb/Cu/Inconel 718 interfacial zone; (**c**) the Cu/Inconel 718 interfacial zone.

Figure 2 illustrates the results of defect analysis in the interfacial zones of the Inconel 718/Ti6Al4V multi-material with the Cu+Nb interlayer. It can be observed that no significant defects are present in the zones of the alloys (Ti6Al4V and Inconel 718) and pure metals (Cu and Nb). The Cu region has a low amount of porosity, which may be related to the low manufacturability of this material, primarily due to its physical properties (high conductivity and high reflectivity). A similar situation is observed in the Nb zone. It is important to highlight the presence of defects in the Cu/Nb interfacial zone. The number of defects is minimal, but it has the potential to impact the mechanical properties. Additionally, the samples exhibit regions of alloys intermixing. This feature is examined in more detail in the subsequent section.

**Figure 3:**
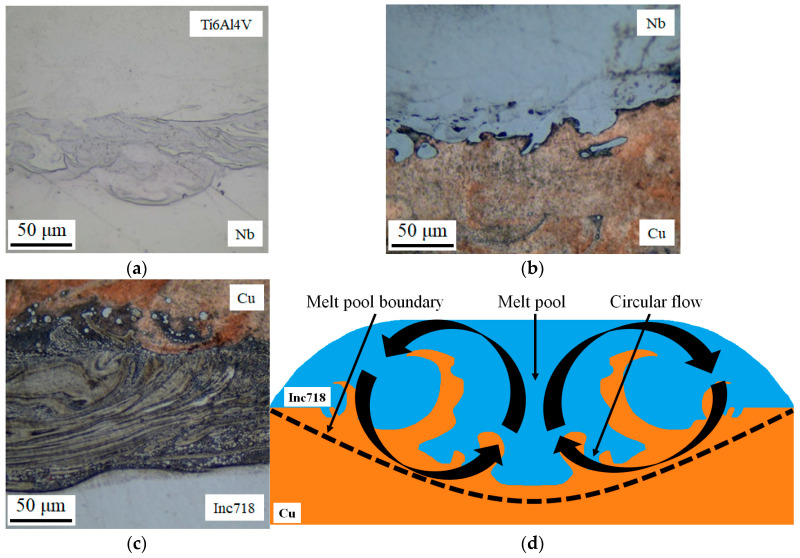
The microstructure analysis in the interfacial zones of the Inconel 718/Ti6Al4V multi-material sample: (**a**) the Ti6Al4V/Nb interfacial zone; (**b**) the Nb/Cu interfacial zone; (**c**) the Cu/Inconel 718 interfacial zone; (**d**) the schematic representation of the Marangoni effect (the bold lines indicate the direction of flow within the melt pool).

Figure 3 illustrates the findings of a microstructure study on the Inconel 718/Ti6Al4V multi-material with a Cu+Nb interlayer. It can be observed that the formation of island macro-segregation (mixing regions) is evident in all interfacial zones. The microstructure of the Ti6Al4V/Nb, Nb/Cu, and Cu/Inconel 718 interfaces was also examined. The formation of Ostwald macro-segregation can be explained by the Marangoni effect [43]. The Marangoni effect occurs when temperature increases in the melt pool cause the surface tension to decrease, thereby facilitating the flow of molten metal in the opposite direction (Figure 3d). The constant inflow of energy serves to enhance the reverse flow, which is then returned to the center of the melt pool, where it forms vortices [44]. As a consequence of the rapid cooling and the insufficient time for the distribution of chemical elements, inhomogeneities emerge, which give rise to the formation of island macro-segregation in vortex flows [45]. This phenomenon is rather negative, since in these zones undesirable phases, such as brittle IMCs, can form [46]. It is reasonable to assume that the amount of island macro-segregation can be reduced by selecting printing parameters with a lower volume energy density. However, it should be considered that too low energy density can lead to the formation of defects (non-melting). To obtain a more accurate understanding of how process parameters influence the formation of island macro-segregation in multi-materials, further studies are required.

### 3.2. Chemical and Phase Composition of the Inconel 718/Ti6Al4V Multi-Material Samples

**Figure 4:**
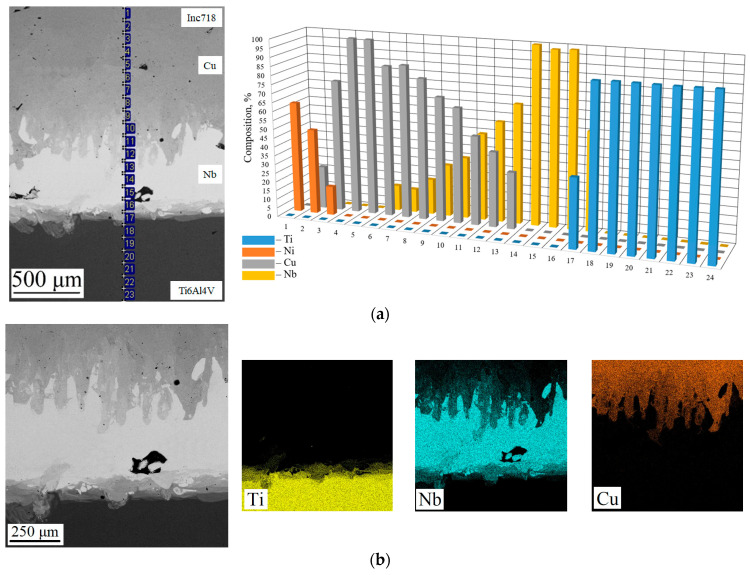
The chemical composition study in the interfacial zones of the Inconel 718/Ti6Al4V multi-material sample: (**a**) chemical element distribution; (**b**) element distribution maps.

Figure 4 shows the chemical composition analysis of the Inconel 718/Ti6Al4V multi-material with the Cu+Nb interlayer. It can be observed that the interfacial zones of Ti6Al4V/Nb and Inconel 718/Cu exhibit a relatively sharp transition in the chemical composition. Here, it is necessary to introduce the concept of an interfacial zone—the zone at the interface of two materials, in which a change in chemical composition is observed (different from the defined composition of the alloy or metal). A sharp transition may be indicative of a narrow interfacial zone. In contrast, the Nb/Cu interfacial zone exhibits a gradual transition from one element to another, which is confirmed by the image of the investigated area (Figure 4a) and the chemical element distribution maps (Figure 4b). The results of the chemical composition study indicate that the width of the Nb/Cu transition zone is approximately 700 μm. The presence of the interfacial zone has a twofold impact: Firstly, it serves to reduce the amount of stress concentration that can occur at a sharp interface between materials. Conversely, the combination of two distinct compositions may result in the formation of defects due to a discrepancy between the chosen parameters and the composition, as well as the formation of undesirable phases.

Figure 4b illustrates the element distribution maps (Ti, Nb, and Cu) in the interfacial zones Ti6Al4V/Nb and Nb/Cu. It can be observed that the Ti6Al4V/Nb interfacial zone exhibits a relatively well-defined element distribution map. The alloy is observed to be mixed with the metal, although the width of this mixing zone is relatively narrow. In contrast, the Nb/Cu interfacial zone exhibits a pronounced mixing of the two metals. It can be observed that the shape of these regions is similar to the shape of the melt pool during the “keyhole” mode [47]. In the Ti6Al4V/Nb and Nb/Cu interfacial zones, blowholes are observed. Such defects may be the result of a discrepancy between the process parameters and the composition, which can occur due to the mixing of metals and alloys. An additional possible explanation is that the parameters for Nb and Cu exhibit sufficiently high values of volume energy density, which may result in an increasing sensitivity to defect formation.

**Figure 5:**
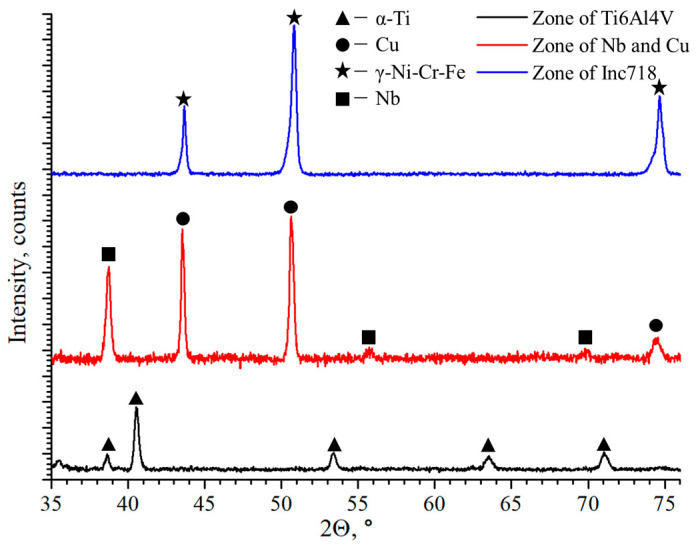
The phase composition study in the interfacial zones of the Inconel 718/Ti6Al4V multi-material sample.

Figure 5 shows the results of the phase composition study in the Inconel 718/Ti6Al4V multi-material with the Cu+Nb interlayer. It can be observed that no new phases occur during the manufacturing process of the multi-material samples. The specified phases are present in the alloy zones and the Cu+Nb interlayer. The interaction of Ti6Al4V and Inconel 718 can lead to the formation of IMCs such as TiNi_3_ and Ti_2_Ni, which can cause embrittlement. However, these phases were not observed, which may indicate either their absence or a minimal presence. It can thus be assumed that the formation of brittle IMCs was probably avoided during the manufacturing process of the multi-material samples. Furthermore, the interaction of Ti6Al4V with Cu can lead to the formation of the brittle IMC CuTi_2_ [48]. Nevertheless, the Nb interlayer between the Ti6Al4V and Cu can inhibit the formation of this IMC.

### 3.3. Microhardness of the Inconel 718/Ti6Al4V Multi-Material Samples

**Figure 6:**
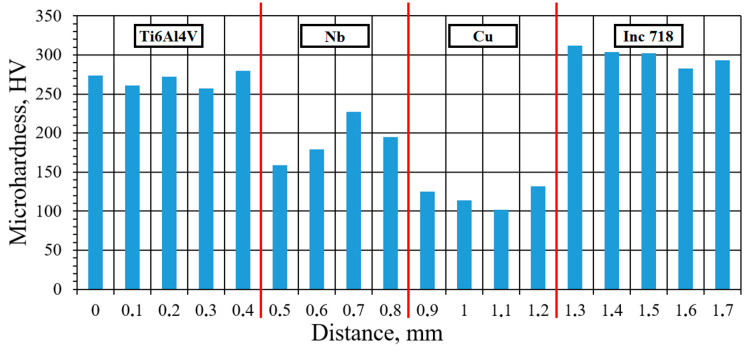
The microhardness analysis in the interfacial zones of the Inconel 718/Ti6Al4V multi-material sample (the red lines indicate the approximate boundaries of the metal and alloy zones).

Figure 6 illustrates the results of the microhardness analysis in the interfacial zones of the Inconel 718/Ti6Al4V multi-material sample with the Cu+Nb interlayer. There are no sudden changes in hardness at transitions from the Ti6Al4V to the Inconel 718. In accordance with the relatively small steps during microhardness measurements (100 µm), the absence of sharp changes suggests that IMCs are either minimal or absent entirely, because IMCs usually have a hardness greater than that of the alloy. The microhardness obtained for the alloy zones corresponds to the average values for the studied alloys. During the transition from the Ti6Al4V zone to the Inconel 718 zone through the Nb and Cu zones, the average microhardness values change as follows: 270 → 190 → 120 → 300 HV. Furthermore, the change in microhardness indicates that the interfacial zone between the alloys is narrow, with no gradient decrease in hardness but, rather, with abrupt transitions. The Nb/Cu interfacial zone is an exceptional case, exhibiting a more gradual transition than the others. This observation aligns with the findings of the chemical composition study, which indicated the presence of a broad interfacial zone.

### 3.4. Room-Temperature Tensile Properties and Fracture Analysis of the Inconel 718/Ti6Al4V Multi-Material Samples

Table 3 presents the findings of the room-temperature tensile properties of the Inconel 718/Ti6Al4V multi-material samples with the Cu+Nb interlayer. It can be observed that the property values exhibit minimal differences among themselves, which suggests that the printing process is characterized by good repeatability. The tensile strength reaches 910 MPa, which is a relatively high value. Nevertheless, this result is below the expected values for the Ti6Al4V and Inconel 718. This may be attributed to the presence of uncontrolled mechanical properties in the interfacial zones and the presence of Cu with low mechanical properties. It is also noteworthy that the yield strength is relatively low, at approximately 140 MPa. It can be assumed that the low yield strength is attributable to the fracture mechanics of the multi-material samples. When the multi-material is undergoing elongation, the first to enter plasticity are Cu and Nb, which have relatively low values of mechanical properties. It may be possible to enhance the properties through the application of heat treatment and the selection of a higher-strength interlayer. Furthermore, the impact of the Cu and Nb layers’ thickness on the mechanical properties can be examined. Additionally, the process parameters in the interfacial zones can be optimized in order to reduce the number and size of island macro-segregations.

**Figure 7:**
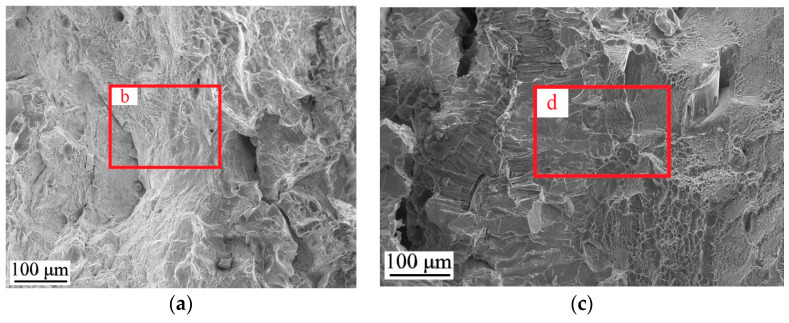

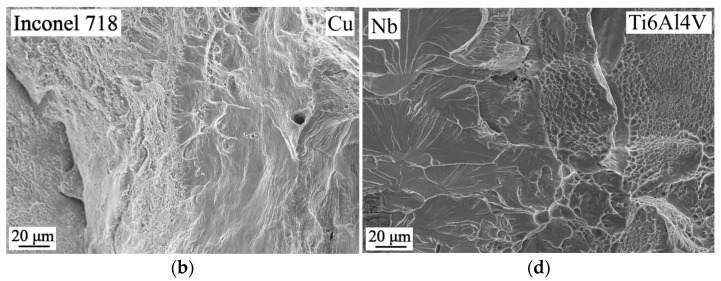
Fracture analysis of the Inconel 718/Ti6Al4V multi-material sample: (**a**,**b**) the Inconel 718/Cu interfacial zone; (**c**,**d**) the Nb/Ti6Al4V interfacial zone.

Figure 7 illustrates the findings of fracture analysis of the Inconel 718/Ti6Al4V multi-material sample with the Cu+Nb interlayers after tensile testing. Two main zones were considered: the Inconel 718/Cu interfacial zone (Figure 7a,b), and the Nb/Ti6Al4V interfacial zone (Figure 7c,d). The Inconel 718 alloy zone is distinguished by the presence of shallow pits and ridges (Figure 7b). Pits are absent in the Cu zone, which is typical for this alloy. However, an inclined shear zone with slip lines is present (Figure 7b). This type of fracture is associated with samples that fracture under shear loading [49]. It can be assumed that the multi-material structure forces Cu to fracture by the shear mechanism. However, additional studies are needed to confirm this hypothesis. In the Nb zone, the metal exhibits an intergranular fracture mechanism, where the fracture has river patterns and a ductile fracture band (Figure 7d). The Ti6Al4V alloy zone is distinguished by the presence of pits of varying sizes, which correspond to the ductile fracture pattern of this alloy (Figure 7d).

## 4. Conclusions

The Inconel 718/Ti6Al4V multi-material is of significant potential for utilization in the aerospace industry. Nevertheless, the fabrication of this multi-material presents certain challenges due to the non-weldability of the two alloys. There is a lack of research regarding the formation of an interlayer in the Inconel 718/Ti6Al4V multi-material obtained by SLM. This highlights the importance of conducting further studies in this area. This study examined the Inconel 718/Ti6Al4V multi-material with the Cu and Nb interlayer produced by SLM. For this purpose, it was necessary to examine the microstructure, the chemical and phase composition, and the hardness in the interfacial zones of the multi-material samples, in order to determine the impact of utilizing interlayers on the mechanical properties of the multi-material samples. The following results were obtained:

1. No significant defects were observed in the zones of the alloys (the Ti6Al4V and the Inconel 718) and pure metals (Cu and Nb). However, such defects were observed in the Cu/Nb interfacial zone. The formation of island macro-segregations was observed in all interfacial zones: Ti6Al4V/Nb, Nb/Cu, and Cu/Inconel 718. The Ti6Al4V/Nb and Inconel 718/Cu interfacial zones exhibited a relatively sharp transition in chemical composition. In contrast, the Nb/Cu interfacial zone exhibited a gradual transition from one element to another. The results of the chemical composition study indicated that the width of the Nb/Cu transition zone was approximately 700 μm. No new phases were observed during the manufacturing process of the multi-material samples. The alloy zones were characterized by the presence of phases that are typical for such materials, as well as for the Cu-Nb interlayer. Thus, it can be assumed that the formation of brittle IMCs was prevented.
2. The microhardness of the alloys and metals under examination correlated with the mean values for these materials. During the transition from the Ti6Al4V zone to the Inconel 718 zone through the Nb and Cu zones, the average microhardness values changed as follows: 270 → 190 → 120 → 300 HV. The change in microhardness values in the transition from alloys to pure metals was abrupt. However, the interfacial zone between Nb and Cu exhibited a more gradual change. The tensile strength of the multi-material samples reached 910 MPa, which is a relatively high value. However, the obtained results were below the properties of Ti6Al4V and Inconel 718. It is also noteworthy that the yield strength was relatively low, at approximately 140 MPa.

## Figures and Tables

**Table 1:** Particle size distribution of powders.

Vol. %	Powders			
	Ti6Al4V	Nb	CuCr1Zr	Inconel 718
	<μm	<μm	<μm	<μm
10	19	47	14	20
50	35	83	29	38
90	63	128	52	66

**Table 2:** The process parameters of SLM.

Materials	Parameters			
	Laser Power	Scanning Speed	Hatch Distance	Layer Thickness
	Watt	mm/s	mm	mm
Ti6Al4V	275	805	0.12	0.05
Nb	275	200	0.1	0.05
CuCr1Zr	250	200	0.1	0.05
Inconel718	275	760	0.1	0.05

**Table 3:** Room-temperature tensile properties of the Inconel 718/Ti6Al4V multi-material samples.

No. of Sample	Properties		
	Yield Strength	Ultimate Tensile Strength	Strain
	MPa	MPa	%
1	145.77	891.72	15.51
2	114.06	915.93	18.91
3	151.71	910.7	13.51
Average	137.18 ± 15.41	906.11 ± 9.6	15.98 ± 1.96

## Data Availability

The original contributions presented in the study are included in the article, further inquiries can be directed to the corresponding author.
